# Impact of the Sensory and Sympathetic Nervous System on Fracture Healing in Ovariectomized Mice

**DOI:** 10.3390/ijms21020405

**Published:** 2020-01-08

**Authors:** Tanja Niedermair, Rainer H. Straub, Christoph Brochhausen, Susanne Grässel

**Affiliations:** 1 Department of Orthopaedic Surgery, Experimental Orthopaedics, Centre for Medical Biotechnology (ZMB/Biopark 1), University of Regensburg, 93053 Regensburg, Germany; tanja.niedermair@ukr.de; 2 Institute of Pathology, University of Regensburg, 93053 Regensburg, Germany; christoph.brochhausen@ukr.de; 3 Laboratory of Experimental Rheumatology and Neuroendocrine Immunology, Department of Internal Medicine I, University of Regensburg, 93053 Regensburg, Germany; rainer.straub@ukr.de

**Keywords:** fracture, substance P, alpha-CGRP, sympathectomy, sympathetic nerve fibers, ovariectomy, sensory nervous system, bone repair, bone remodeling

## Abstract

The peripheral nervous system modulates bone repair under physiological and pathophysiological conditions. Previously, we reported an essential role for sensory neuropeptide substance P (SP) and sympathetic nerve fibers (SNF) for proper fracture healing and bone structure in a murine tibial fracture model. A similar distortion of bone microarchitecture has been described for mice lacking the sensory neuropeptide α-calcitonin gene-related peptide (α-CGRP). Here, we hypothesize that loss of SP, α-CGRP, and SNF modulates inflammatory and pain-related processes and also affects bone regeneration during fracture healing under postmenopausal conditions. Intramedullary fixed femoral fractures were set to 28 days after bilateral ovariectomy (OVX) in female wild type (WT), SP-, α-CGRP-deficient, and sympathectomized (SYX) mice. Locomotion, paw withdrawal threshold, fracture callus maturation and numbers of TRAP-, CD4-, CD8-, F4/80-, iNos-, and Arg1-positive cells within the callus were analyzed. Nightly locomotion was reduced in unfractured SP-deficient and SYX mice after fracture. Resistance to pressure was increased for the fractured leg in SP-deficient mice during the later stages of fracture healing, but was decreased in α-CGRP-deficient mice. Hypertrophic cartilage area was increased nine days after fracture in SP-deficient mice. Bony callus maturation was delayed in SYX mice during the later healing stages. In addition, the number of CD 4-positive cells was reduced after five days and the number of CD 8-positive cells was additionally reduced after 21 days in SYX mice. The number of Arg1-positive M2 macrophages was higher in α-CGRP-deficient mice five days after fracture. The alkaline phosphatase level was increased in SYX mice 16 days after fracture. Absence of α-CGRP appears to promote M2 macrophage polarization and reduces the pain threshold, but has no effect on callus tissue maturation. Absence of SP reduces locomotion, increases the pain-threshold, and accelerates hypertrophic callus tissue remodeling. Destruction of SNF reduces locomotion after fracture and influences bony callus tissue remodeling during the later stages of fracture repair, whereas pain-related processes are not affected.

## 1. Introduction

A network of sensory and sympathetic nerve fibers can be observed throughout bone tissue, innervating cortical and trabecular bone, the bone marrow, and the periosteum. Neurotransmitters from these nerve fibers are critically involved in the control of vascularization, bone development, and bone growth. In the adult skeleton, they modulate bone cell function and differentiation as well as bone remodeling processes [1,2]. In addition, neurotransmitter signaling has been shown to influence bone regeneration during fracture healing [3] and is involved in cartilage and subchondral bone alterations during disease progression of osteoarthritis [4].

The sensory neuropeptide substance P (SP) is synthesized from the tachykinin 1 (*TAC1*) gene and signals predominantly via the neurokinin (NK) 1 receptor [5,6]. Recent studies have discovered a strong neuro-osteogenic connection. Sensory nerve fibers demonstrated intense nerve regeneration and fast regrowth (sensory sprouting) into injured tissue after tibial fractures [7]. Our group reported increased pressure resistance and impaired bone structure after fracture in SP-deficient mice. In addition, callus maturation was altered [4]. Ding et al. described poorer fracture healing and a decrease of SP at the injury site after femoral shaft fractures in ovariectomized (OVX) mice compared to control mice [8], suggesting a critical role of this neuropeptide during bone repair, not only under physiological conditions, but also under pathophysiological conditions.

α-Calcitonin gene-related peptide (α-CGRP) is synthesized from the *CALC I* gene by alternative splicing and signals preferentially via the calcitonin receptor-like receptor (CLR)/receptor activity modifying protein (RAMP) 1 complex [9]. The binding of α-CGRP to this receptor complex located on osteoblasts, bone marrow macrophages (BMM), and osteoclasts under in vitro conditions mainly results in anabolic effects promoting bone formation [10,11]. In vivo, this osteo-anabolic effect has been shown in a rat-based bone fatigue model by increasing osteogenesis and inhibiting osteoclastic bone resorption [12]. In a mouse model, deficiency of α-CGRP resulted in the development of an age-related osteopenic bone phenotype [13]. These effects point out critical regulatory functions of α-CGRP during bone remodeling processes and let us assume a likewise important function during bone repair.

Catecholamines, such as epinephrine and norepinephrine (NE), are the major neurotransmitters of the sympathetic nervous system (SNS). By binding to different adrenergic receptor subtypes (α1, α2, β1, β2, β3) expressed on a variety of bone cells, the SNS is critically involved in modulating bone homeostasis and remodeling processes [14,15,16,17]. Disorders, such as depression and drugs that exert their effects via adrenergic receptors have been associated with increased bone loss and fracture risk [18,19]. In previous studies, we analyzed bone properties and fracture healing in chemically sympathectomized (SYX) mice. The destruction of peripheral sympathetic nerve fibers through the injection of 6-hydroxydopamine (6-OHDA) reduces the production of adrenergic neurotransmitters by about 80% [20,21] and resulted in impaired structural bone properties, which was presumably caused by increased bone resorption. Additionally, the absence of sympathetic innervation delayed callus maturation during early tibial fracture healing [4]. Altogether, the SNS seems to have a crucial impact on physiological bone remodeling. However, its role in fracture healing and bone regeneration under impaired musculoskeletal conditions is not well known.

A number of comorbidities exist which impact on bone homeostasis and often result in fracture-healing complications, such as ischemia, impaired vascularization, and osteoporosis [22]. Estrogen belongs to the gonadocorticoid class of steroid hormones and plays a key role in the growth, regulation, and maintenance of bone mass. Besides aging and inactivity, estrogen deficiency counts as a major cause of involutional osteoporosis. The decrease in estrogen levels at and after menopause results in increased bone turnover rates and remodeling imbalances [23,24], which also impairs the process and the outcome of fracture healing. 

As indicated above, neurotransmitter signaling has crucial modulatory functions during bone remodeling and repair processes. Nevertheless, it is unknown whether neurotransmitters of the sensory and sympathetic nervous system have a similar modulating effect under impaired conditions as they do have under physiological musculoskeletal conditions. Therefore, we used ovariectomized (OVX) mice as a model for osteoporosis, resembling the human menopausal situation, for studying fracture healing. The aim of the study was to delineate the role of the sensory and sympathetic nervous system and their neurotransmitters in bone regeneration under pathophysiological conditions. We compared fracture healing in OVX SP- and α-CGRP-deficient mice and in OVX mice after chemical destruction of the SNS (SYX) with fracture healing in OVX WT controls.

## 2. Results

### 2.1. Locomotion Analysis before and after Fracture

**Figure 1:**
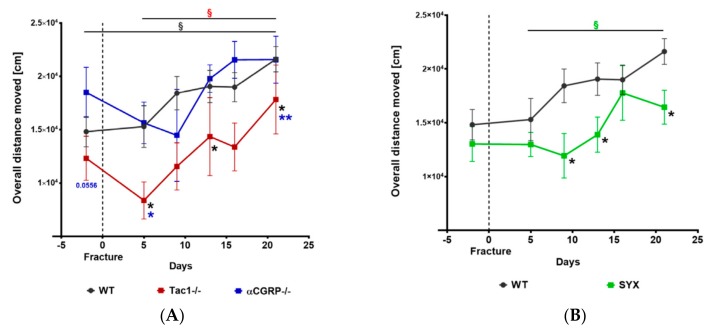
Time course of locomotion analysis before and after fracture. Nightly locomotion of Tac1−/−, α-CGRP−/− (**A**), SYX (**B),** and WT (**A**,**B**) mice before and after fracture was analyzed during a 1 h time span in separate cages. Overall distance moved was monitored with the EthoVision XT 9 Software. Data are expressed as mean ±  SEM. § indicates significance within the respective groups, demonstrated by the respective color. * in black indicate differences between neurotransmitter deficient mice and WT animals. */**/*p*-Values in blue indicate differences between Tac1−/− and α-CGRP−/− mice. * = *p* ≤ 0.05; ** = *p* ≤ 0.01. N (sample number) = 5–8.

Differences in weight bearing during fracture healing might influence the healing process. Therefore, we monitored nightly locomotion during a time span of 1 h. The overall distance moved did not differ between α-CGRP−/− and WT mice but we observed reduced locomotion of Tac1−/− mice before and 5, 13, and 21 days after setting the fractures (Figure 1A). Likewise, locomotion was reduced in SYX mice 5, 9, and 21 days after fracture when compared to WT control animals (Figure 1B).

### 2.2. Mechanical Hyperalgesia before and after Fracture

**Figure 2:**
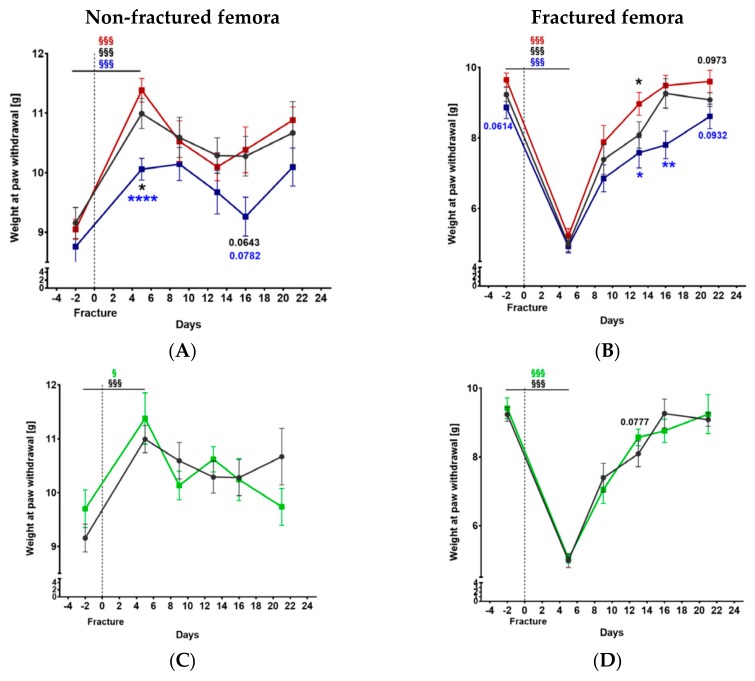
The time course of mechanical hyperalgesia before and after fracture. The withdrawal threshold was measured using the Dynamic Plantar Aesthesiometer test for the non-fractured (**A**,**B**) and the fractured (**C**,**D**) femora of Tac1−/−, α-CGRP−/− (**A**,**C**), and SYX (**B**,**D**) mice in comparison to WT (black) mice. Data are expressed as mean ± SEM. §/§§§ indicates significance within the respective groups, demonstrated by the respective color. */*p*-values in black indicate differences between neurotransmitter deficient mice and WT animals. */**/****/*p*-Values in blue indicate differences between Tac1−/− and α-CGRP−/− mice. * = *p* ≤ 0.05; ** = *p* ≤ 0.01; **** = *p* ≤ 0.0001. N = 6–14.

Weight at paw withdrawal increased significantly in the non-fractured hind limbs 5 days after fracture in all groups (Figure 2). The withdrawal threshold measured for non-fractured femora of α-CGRP−/− mice was lower on day 5 and tended to be lower on day 16 compared to Tac1−/− and WT mice (Figure 2A). No difference was observed between SYX and WT mice (Figure 2B). For the fractured femora, withdrawal threshold dropped 5 days after fracture in all groups (Figure 2), indicating mechanical hyperalgesia shortly after fracture setting but was back to normal levels at day 21. Similar to the results for the non-fractured femora, the weight at paw withdrawal was reduced in fractured femora of α-CGRP−/− mice before and on days 13, 16, and 21 (by trend) after fracture when compared to Tac1−/− mice. In addition, the withdrawal threshold was significantly increased at 13 days and tended to increase after 21 days in the fractured legs of Tac1−/− compared to WT mice (Figure 2C). The withdrawal threshold did not differ between SYX and WT mice (Figure 2D).

### 2.3. Maturation of Mesenchymal, Cartilaginous and Bony Callus Tissue

**Figure 3:**
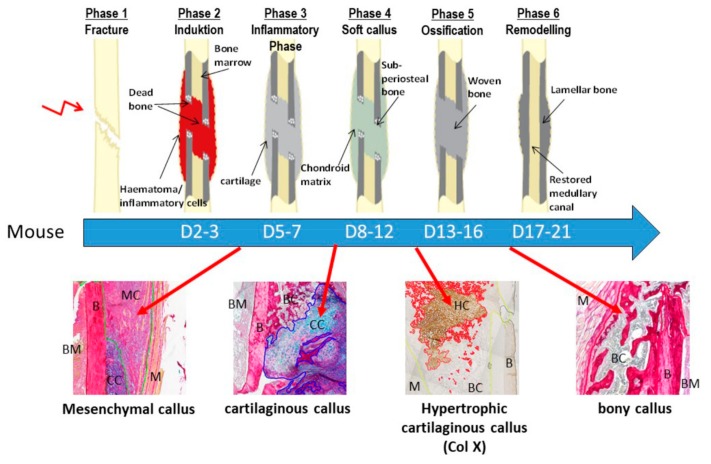
A representative figure showing the different murine fracture healing phases. Murine fracture healing phases range from initial fracture to remodeling of the bony callus. The time line is shown under the scheme which depicts the healing phases. Images under the time line demonstrate the different tissue types typically present during callus maturation until day 21. The mesenchymal callus tissue accounts for the major part of the callus during the early healing phases (Phase 2/3). Around 8–12 days after fracture, the major part of the callus consists of cartilaginous callus tissue. The cartilaginous matrix turns hypertrophic and becomes mineralized and is most present around day 13. Finally, during the remodeling phase (days 17–21) the major part of the callus tissue consists of trabecular bony callus tissue. D = day; BM = bone marrow; B = bone; MC = mesenchymal callus; M = muscle; CC = cartilaginous callus; BC = bony callus; HC = hypertrophic callus. Black arrows point to the specific tissue in the fracture healing scheme. Red arrows define the time points, where the tissue types in the images under the scheme are most prominent during the time course of fracture healing in the mouse. Images where scanned with 10x magnification (TissueFaxSi plus System, Tissue Gnostics).

The different phases of endochondral fracture healing are highly distinguishable by the different callus tissue types that are most prominent at specific time points (Figure 3).

**Figure 4:**
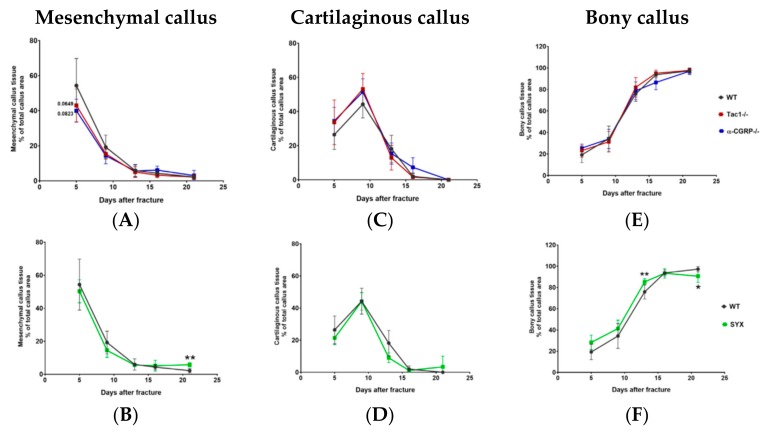
Callus maturation during fracture healing. Area of mesenchymal (**A**,**B**), cartilaginous (**C**,**D**) and bony (**E**,**F**) callus tissue during fracture healing in Tac1−/−, α-CGRP−/− (**A**,**C**,**E**), SYX (**B**,**D**,**F**), and WT mice (**A**–**F**) at the time points 5, 9, 13, 16, and 21 days after fracture. Values were calculated as a percentage of total callus area and are presented as mean ± SD. */**/*p*-Values in black indicate differences between neurotransmitter deficient and SYX mice to WT. * = *p* ≤ 0.05; ** = *p* ≤ 0.01. N = 4–7.

Shortly after fracture, mesenchymal callus tissue accounted for the major part of total callus tissue and decreased steadily over time until day 21, the observation end point of this study. The amount of mesenchymal tissue tended to be lower 5 days after fracture in Tac1−/− and α-CGRP−/− mice compared to WT mice, but no further differences were observed (Figure 4A). Mesenchymal callus showed similar tissue maturation in SYX and WT mice until day 16 but the region of mesenchymal callus remained significantly larger in SYX mice thereafter (Figure 4B).

The main peak of cartilaginous callus tissue was observed around 9 days after fracture, followed by a continuous decrease until day 21. This time line was similar in all mice groups (Figure 4C,D).

The area of bony callus tissue increased from day 5 to day 21 after fracture. The time course of ossification was comparable in Tac1−/−, α-CGRP−/−, and WT mice (Figure 4E). In contrast, the area of bony callus tissue was larger 13 days after fracture in SYX compared to control mice. However, from day 16 on bony callus maturation was delayed in SYX mice until the observation end point (Figure 4D).

### 2.4. Hypertrophic Callus Area and Number of TRAP-Positive Callus Cells

**Figure 5:**
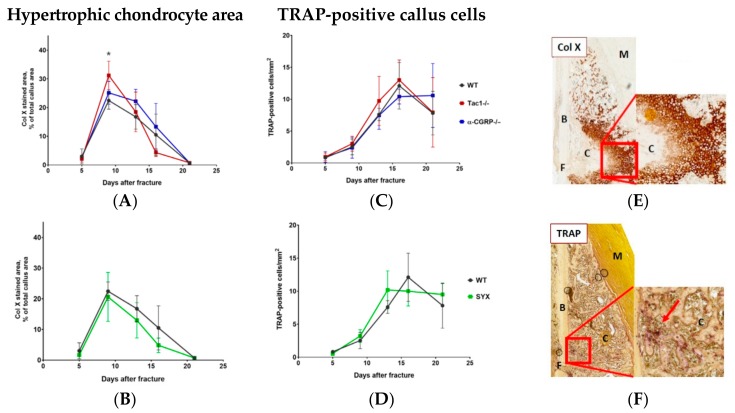
Hypertrophic cartilage area and number of TRAP-positive callus cells**.** (**A**,**B**) Number/area of hypertrophic chondrocytes, visualized by collagen X staining, in Tac1−/−, α-CGRP−/− (**A**), SYX (**B**), and WT mice (**A**,**B**) at the time points 5, 9, 13, 16, and 21 days after fracture. Values were calculated as a percentage of total callus area and are presented as mean ± SD. * in black indicates differences between neurotransmitter deficient mice and WT animals. (**C**,**D**) Number of TRAP-positive cells in the callus tissue of Tac1−/−, α-CGRP−/− (**C**), SYX (**D**), and WT mice (**C**,**D**) at the time points 5, 9, 13, 16, and 21 days after fracture. The number of cells was calculated in relation to the total area of callus tissue as number/mm^2^. (**E**) Representative image of collagen X stained hypertrophic callus area of an α-CGRP−/− mouse 13 days after fracture. (**F**) Representative image of TRAP-positive callus cells 16 days after fracture in the callus tissue of a WT mouse. Overview images scanned with 20× magnification (TissueFAXSi plus). Red boxes demonstrate the enlarged view. Red arrow points to stained cells. Col X = collagen X; B = bone, C = Callus tissue, F = Fracture site, M = Muscle. * = *p* ≤ 0.05. N = 4–6.

Hypertrophic chondrocyte differentiation with subsequent cartilaginous matrix mineralization is a key process during endochondral fracture healing. The highest number of collagen X stained hypertrophic chondrocytes was counted 9 days after fracture and decreased constantly until day 21. Hypertrophic cartilage region was larger in Tac1−/− mice compared to WT mice 9 days after fracture (Figure 5A). Maturation of hypertrophic callus tissue was similar in α-CGRP−/− (Figure 5A), SYX (Figure 5B), and WT mice (Figure 5A,B).

Likewise, number of Tatrate-resistent Acid Phosphatase (TRAP)-positive callus cells was comparable throughout fracture healing in all groups (Figure 5C,D). The highest numbers were observed in all groups around 16 days after fracture.

Figure 5E,F (and Supplementary Figure S1) show representative images of collagen X (Figure 5E) and TRAP (Figure 5F) stained callus tissue sections.

### 2.5. Number of CD4- and CD8-Positive T Cells during Callus Maturation

**Figure 6:**
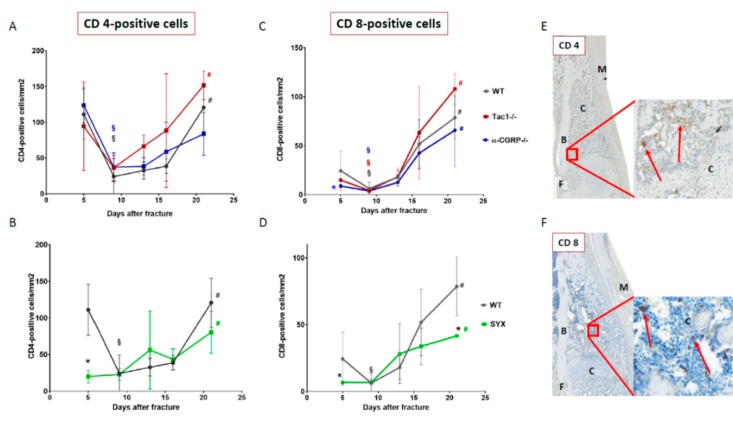
Number of CD4- and CD8-positive cells during callus maturation. Number of CD4-positive helper T cells (**A**,**B**) and CD8-positive cytotoxic T cells (**C**,**D**) in the callus tissue of Tac1−/−, α-CGRP−/− (**A**,**C**), SYX (**B**,**D**), and WT mice (**A**–**D**) at the time points 5, 9, 13, 16, and 21 days after fracture. Number of cells was calculated in relation to the amount of callus tissue as number/mm^2^. (**E**) Representative image of CD 4-positive helper T cells 13 days after fracture in the callus tissue of a Tac1−/− mouse. (**F**) Representative image of CD 8-positive cytotoxic T cells 16 days after fracture in the callus tissue of a WT mouse. Overview images scanned with 20x magnification (TissueFAXSi plus). Red boxes demonstrate the enlarged view. Red arrow points to stained cells. B = bone, C = Callus tissue, F = Fracture site, M = Muscle. § indicates significance between days 5 and 9 within the respective groups and # indicates significance between days 9 and 21 within the respective groups, demonstrated by the respective colors. * in black indicates differences between neurotransmitter deficient mice and WT animals. * in blue indicates differences between Tac1−/− and α-CGRP−/− mice. */§/# = *p* ≤ 0.05. N = 3–4.

CD4-positive helper T cells were abundantly present in the callus tissue of WT, α-CGRP−/−, and Tac1−/− mice 5 days after fracture. In all groups, the number of CD4-positive cells decreased markedly around day 9 but increased again towards the remodeling time point 21 days after fracture (Figure 6A). The number of CD4-positive helper T cells was reduced in the callus tissue of SYX compared to WT mice 5 days after fracture (Figure 6B). No differences were detected at later time points as the number of CD4-positive cells increased slowly in the callus tissue of SYX and WT mice until day 21 (Figure 6B).

The number of CD8-positive cytotoxic T cells was lower in α-CGRP−/− compared to Tac1−/− mice 5 days after fracture. Afterwards, cell number increased steadily and comparably throughout the fracture healing process until day 21 in all mice groups (Figure 6C,D). At day 5 after fracture, the number of CD8-positive T cells was lower in SYX mice compared to WT controls. From day 5 onwards, the number of CD8-positive T cells increased in the callus tissue of SYX mice throughout callus differentiation process but remained significantly lower on day 21 compared to WT (Figure 6D).

Figure 6E,F (and Supplementary Figure S1) show representative images of CD4- and CD8-positive cells in the callus tissue. We detected CD4- and CD8-positive cells in the mesenchymal callus and in the bony callus tissue. No CD4- or CD8-positive cells were observed in the cartilaginous soft callus and the hypertrophic cartilaginous callus area.

### 2.6. Macrophage Subtype Profile Five Days after Fracture

**Figure 7:**
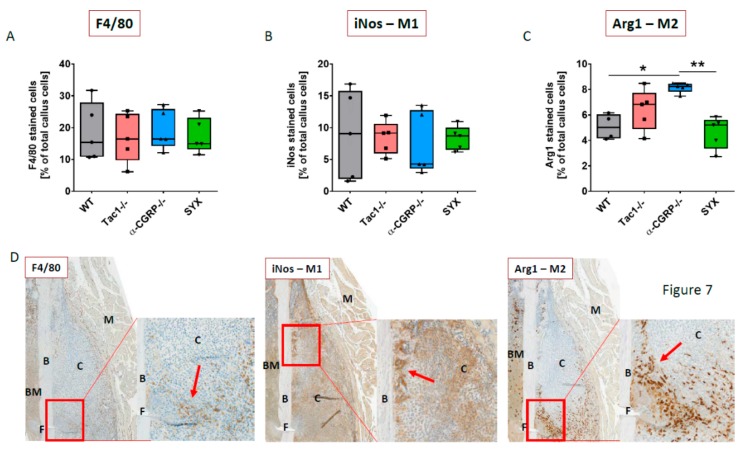
Macrophage subtype profile during the early fracture healing phase. Macrophages in the callus tissue of WT, Tac1−/−, α-CGRP−/−, and SYX mice 5 days after fracture were detected using F4/80 as an overall macrophage marker (**A**), inducible nitric oxide synthase (iNos) as a marker for M1 macrophages (**B**), and Arginase 1 (Arg1) as a marker for M2 macrophages (**C**). Values are calculated as a percentage of total callus cells and are shown as median ± Min/Max. (**D**) Representative images of the F4/80, iNos, and Arg1 staining in the callus tissue of a Tac1−/− mouse. Red boxes demonstrate the enlarged view. Red arrow points to stained cells. Magnification: 20× (TissueFAXSi plus). B = bone, C = Callus tissue, BM = bone marrow, F = Fracture site, M=Muscle. * = *p* ≤ 0.05, ** =*p* ≤ 0.01. N = 5.

Macrophage subtypes M1 and M2 determine the fracture healing process during the early inflammatory healing phase. Staining for F4/80 as an overall macrophage marker revealed no differences (Figure 7A). Likewise, the percentage of iNos stained M1 macrophages did not differ between the 4 groups (Figure 7B). A similar number of Arg1 stained M2 macrophages was observed in Tac1−/−, SYX and WT mice, whereas more Arg1-positive M2 macrophages were counted in the callus tissue of α-CGRP−/− compared to WT and SYX mice (Figure 7C). Representative images of M1 (iNos-positive) and M2 (Arg1-positive) macrophage subtypes are shown in Figure 7D (and Supplementary Figure S1).

F4/80-positive cells were abundantly expressed in the mesenchymal callus tissue and occasionally in regions, where cartilage differentiation was still at the beginning. We also detected F4/80-positive cells in the bony callus tissue, which started to differentiate via intramembranous ossification at the proximal and distal ends of the callus, but not in mature cartilage or in hypertrophic cartilage tissue. Likewise, deeply brown stained, iNos-positive cells were observed in mesenchymal and bony callus tissue. We also found iNos staining in non-hypertrophic cartilage and hypertrophic cartilage callus areas. This staining was less intense and could clearly be distinguished from the dark brown stained cells. The staining in the non-hypertrophic cartilage and hypertrophic cartilage areas originated from chondrocytes, which have been shown to produce iNos [25,26]. Therefore, these cells were subtracted from the dark brown stained cells during analysis. Arg1-positive cells were abundantly expressed in the mesenchymal callus tissue and were prominently located near the fracture gap, whereas only a small number of Arg1-positive cells was detected in the bony callus tissue. No Arg1-positive staining was detected in non-hypertrophic cartilage and hypertrophic cartilage.

### 2.7. Serum Analysis of Bone Resorption and Bone Formation Markers

Remodeling markers in the serum were assayed at time points where remodeling processes are important for the progress of fracture healing. Serum analysis revealed no differences between CTX I concentration (pg/mL) in the serum of WT, Tac1−/−, α-CGRP−/−, and SYX mice at the time points 9and 16 days after fracture (Table 1). In addition, serum ALP activity was similar in all groups 9 days after fracture. At the later time point, 16 days after fracture, serum ALP activity tended to be higher in SYX compared to WT mice, whereas ALP activity was comparable between Tac1−/−, α-CGRP−/−, and WT mice (Table 1).

## 3. Discussion

The healing of bone fractures is a complex process including a wide range of different cell types and its temporal sequence is strictly regulated [27]. Beside fracture stability and blood supply, the innervation by sensory and sympathetic nerve fibers and neurotransmitter signaling play a crucial role during the healing process [4,7,28,29]. So far, most studies have analyzed the neuronal effects on fracture healing under physiological conditions of the musculoskeletal system, meaning the modulating effects of the nervous system on fracture healing under pathological conditions are still unknown. Here, we used the model of ovariectomy in mice—as this model resembles the human menopausal situation—to study the role of sensory and sympathetic neurotransmitters on fracture healing under pathological musculoskeletal conditions.

The biomechanical situation at the fracture site is a crucial factor during callus formation and determines the healing outcome. A huge periosteal callus is formed after early weight bearing of flexible fixed fractures, which results in delayed healing and reduced quality of newly formed bone [30,31]. Pain-related processes influence weight bearing during the healing process and can be analyzed by measuring pressure resistance of the paws of the fractured legs. SP is critically involved in nociception and mediates pain behavior after fracture [32]. In a previous study, we reported increased pressure resistance in Tac1−/− mice during the early healing phase under physiological conditions [4]. Here, the withdrawal threshold was increased in OVX Tac1−/− mice during later healing stages. No differences were measured shortly after fracture, where the withdrawal threshold strongly decreased but was comparable to control mice. This reaction might be due to a reduction of nerve fiber density in the bone after OVX, which was demonstrated in a study with rats [33]. This phenomenon possibly also modulates pain sensation during early fracture healing in control mice, when increased sensory nerve sprouting takes place under physiological healing conditions [7]. Furthermore, reduced pain-sensation led to a higher locomotion activity, in contrast locomotion activity was reduced before and after fracture in Tac1−/− mice. Taken this into account, we assume that Tac1−/− mice put more weight onto the fractured leg during movement but are generally less active, possibly due to behavioral deficits. SP also plays a major role in the central nervous system, and it was linked to sickness behavior and depression [34].

Interestingly, the withdrawal threshold was decreased not only in fractured, but also in non-fractured legs from α-CGRP−/− compared to Tac1−/− and partly compared to WT animals. α-CGRP is widely distributed in nociceptive pathways and might be involved as a neuromodulator in pain transmission [35]. The increased touch sensitivity may be a result of the higher SP concentration, which we detected in the serum of α-CGRP−/− mice, thereby modulating pain-related processes [5]. Despite these differences in touch sensitivity, the overall distance moved was comparable between α-CGRP−/− and WT mice. Therefore, α-CGRP−/− mice might put less weight onto the fractured leg, while they are similarly active compared to WT mice.

Under physiological conditions, SYX increased touch sensitivity 8 days after fracture [4], whereas the withdrawal threshold was comparable between SYX and WT mice in the present study. Thus, the SNS might be involved in pain transmission under certain circumstances, e.g., during abnormal sprouting of sympathetic fibers, or under pain conditions as the complex regional pain syndrome (CRPS), where a sympathetic blockade is the recommended treatment [36,37]. However, it seems to have no effects on pain-related processes during fracture healing under impaired musculoskeletal conditions. Despite this, absence of the SNS affects locomotion activity, as the overall distance moved by SYX mice was reduced after fracture compared to WT mice. The absence of sympathetic signaling might shift the balance to a more parasympathetic influence with a possibly sedative effect on the animals. On the other hand, chemical destruction of SNF with 6-OHDA might impair motivational factors and mechanisms that are involved in exercise behavior, as suggested by Derevenco and colleagues [38].

A study of Ding et al. demonstrated a dramatic decrease of SP in the callus tissue of OVX mice throughout fracture healing, suggesting a crucial neuronal regulatory role [8]. In this study, mesenchymal callus tissue was slightly reduced in Tac1−/− mice during the early healing phase, which was not observed in our earlier studies under physiological conditions [4]. During the middle and late healing phases, the mesenchymal callus tissue of Tac1−/− mice decreased comparable to WT mice. Likewise, the maturation of cartilaginous soft callus and bony callus tissue was comparable. Yet, the area of hypertrophic cartilage tissue was increased in Tac1−/− mice during the middle healing stage, 13 days after fracture. This is in contrast to our findings under physiological conditions, where the area of hypertrophic chondrocytes was clearly decreased at this time point, suggesting a delay in hypertrophic chondrocyte differentiation [4]. In vitro, daily stimulation of chondrocytes with SP for 1 week in a pellet culture system had no effect on matrix components such as collagen X or proteoglycans, but did increase the gene expression of matrix metalloproteinase (*MMP*) 13 on day 14, indicating an influence of SP on matrix remodeling processes [17]. SP is produced by cells in the mesenchymal and cartilaginous callus tissue, but was not detectable in hypertrophic callus tissue [4]. Yet, hypertrophic chondrocytes in the fracture callus express the receptor for SP, neurokinin 1 (NK1R) and, thus, are able to respond to SP stimuli. Therefore, SP seems to play an active role during the remodeling process, specifically of hypertrophic callus tissue. Our results let assume that this role is different under physiological and pathological conditions.

In an in vitro study, we demonstrated that the absence of SP reduced the number of osteoclast-precursor cells and the differentiation capacity to mature osteoclasts, but no differences were observed in osteoclast number in untreated bones of Tac1−/− compared to WT mice [39]. We counted less osteoclasts/mm^2^ in the callus tissue of Tac1−/− mice 13 days after fracture under physiological conditions [4], whereas the present observations revealed no differences between OVX Tac1−/− and WT mice throughout the fracture healing process, strengthening the assumption that SP acts differently under physiological conditions, and that estrogens markedly influence SP effects. It is noteworthy that different fracture models and animal sex were used in these studies. Gender specific hormones and differences in biomechanical forces, due to different stabilization methods, might additionally account for the aforementioned differences.

The absence of sympathetic nerve fibers had no effect on maturation of mesenchymal and cartilaginous callus tissues of SYX mice during the early (day 5) and middle (day 9) healing phases. Interestingly, the early healing phase was affected in SYX mice under physiological conditions, as destruction of sympathetic nerve fibers increased mesenchymal callus and reduced the portion of cartilage tissue at 5 days after fracture. In addition, number of osteoclasts was increased [4]. Under pathological conditions, the absence of sympathetic nerve fibers delayed bony callus development in SYX mice from day 13 until day 21, but had no influence on osteoclast number throughout callus differentiation.

In contrast to WT mice, the serum level of ALP was higher in SYX mice 16 days after fracture, whereas no differences were detectable when analyzing CTX I concentration as a serum marker for bone resorption. Komnenou et al. analyzed ALP serum concentration throughout fracture healing in dogs and compared it with radiological outcomes until complete reunion [40]. Serum ALP increased after fracture and remained increased in the delayed group compared to the normal union group. In a clinical study, similar results were reported indicating that ALP serum concentrations seem to correlate with fracture healing [41]. ALP concentrations remained higher in the delayed healing group, indicating ALP as a marker for critical fractures. Together, these data suggest a critical function of the SNS on bony callus maturation during the late stage of fracture healing under quasi menopausal conditions. The differences in locomotion activity from day 9 post-fracture onwards might contribute to the delay in bony callus maturation.

The formation of a hematoma during the inflammatory phase are critical steps during the early healing process of a fracture and involve the action of macrophages and T-cells [42]. Könnecke et al. described lymphocyte infiltration into the callus in two migration waves. T cells were highly present during the early healing phase and did not infiltrate the cartilaginous tissue with ongoing callus maturation, but were still detectable in the endosteal tissue [43]. Then, concomitant with bony callus development, the number of T-cells increased strongly. This is in line with our study, as we found high numbers of CD4-positive helper T-cells in the callus tissue of Tac1−/−, α-CGRP−/− and WT-mice 5 days after fracture. The number of CD4-positive helper T-cells decreased in all 3 groups during the soft callus phase and increased again later during bony callus formation, suggesting no influence of SP and α-CGRP on helper T-cells during fracture repair in OVX animals.

Shortly after fracture, CD8-positive cytotoxic T-cells were clearly detectable in the callus tissue of WT, Tac1−/−, and—to a slightly smaller extent—in α-CGRP−/− mice. However, CD4-positive helper T-cells outnumbered CD8-positive cytotoxic T-cells (with a ratio of 150:100 cells per mm^2^). This is in accordance with the literature, where it was described that traumatic injuries change the ratio of CD8/CD4-positive cell sunder physiological conditions. CD8-positive cells account for a higher percentage, but CD4-positive cells surpass the number of CD8-positive cells after injuries, as observed in this study shortly after fracture [44]. After the initial phase, the number of CD8-positive cytotoxic T-cells decreased within each of the 3 groups at 9 days after fracture, but increased simultaneously until day 21. This observation suggests that SP and α-CGRP have no impact on cytotoxic T-cell invasion during fracture healing under low estrogen conditions.

In contrast, absence of sympathetic nerve fibers strongly reduced the number of hematoma-derived CD4-positive helper T-cells and CD8-positive cytotoxic T-cells 5 days after fracture. While helper T-cell numbers increased compared to WT from day 9 until day 21, the number of cytotoxic T-cells was reduced in SYX mice 21 days after fracture. During this second invasion phase, T-cells invade the callus via its inner vasculature [43]. Our data indicate that the SNS affects the CD8/CD4 ratio before and during fracture healing. Depletion of CD8-positive T-cells normally favors the healing outcome of a wound [45]. However, CD4-positive T-cells could positively affect bone remodeling through osteoprotegerin production [46]. As a result, the missing effect of CD4-positive cells during the early healing phase in SYX mice might contribute to the delay in bony callus maturation.

Different macrophage subtypes are necessary for successful fracture repair due to participate on in the coordination of both, inflammatory processes and bone anabolic outcomes. Depletion of macrophages at the time of fracture surgery abrogated callus formation in a murine fracture model [47]. The classically activated, iNos expressing M1 macrophages differentiate in response to interferon-γ (IFN-γ) and lipopolysaccharide (LPS), which is produced by a subtype of CD4-positive helper T cells known as Th1 cells or CD8-positive T-cells. The alternatively activated, Arg1 expressing M2 macrophages—which are also defined as wound-healing macrophages—are generated by interleukin-4 (IL-4) mediated signals, which are produced by CD4-positive Th2-helper T cells [48,49]. Schlund et al. reported that the balance between the different macrophage subtypes M1 and M2 changed during fracture healing [50]. During the initial healing period until day 3, the predominantly proinflammatory M1 subtype switched to the more heterogeneous group of the M2 subtype. In addition, the induction of a M2/Th2 phenotype at the fracture site increased bone formation after 21 days. This demonstrates that a shift in the balance can impact on the healing outcome. In our study, neither loss of SP and α-CGRP nor the absence of sympathetic nerve fibers affected the overall macrophage number and the number of iNos-positive, proinflammatory M1 macrophages during the early healing period under impaired musculoskeletal conditions. However, we counted higher percentages of Agr1-positive M2 macrophages in the callus tissue of α-CGRP−/− mice 5 days after fracture. The “anti-inflammatory, pro-regeneration and pro-repair” M2 macrophage subtype is proposed to increase mesenchymal stem cell differentiation and osteoblast function [51,52]. This might also point to a difference in the balance of CD4-positive T cells, namely to increased Th2 T cell numbers, and possibly to a pro-anabolic effect on fracture healing in the absence of α-CGRP. Interestingly, we did not detect differences in callus tissue maturation, in number of bone resorbing cells, or in serum markers for bone resorption and bone formation throughout all of the healing phases. In our study, macrophage subtypes were analyzed 5 days after fracture, but were not investigated at later time points. As the number of CD4-positive T cells failed to increase significantly in the callus tissue from α-CGRP−/− mice from day 9 to 21 while the number of CD8-positive T cells did increase during this time, the ratio of macrophage subtypes might change again during later time points, repealing the effect on M1/M2 subtype ratio during the early healing stage. In total, our data suggest that loss of α-CGRP has no or only a minor impact on fracture callus maturation under impaired conditions due to low estrogen levels. The direct effect of the absence of α-CGRP on the different subtypes of macrophages and T cells during bone repair processes needs to be addressed in further studies.

This study has some limitations. The age of the mice at the time point of OVX might be regarded as too young and the time span between OVX and the time point of fracture might be regarded as too short. However, the changes in bone turnover rate due to OVX are clearly visible in 8 and 12 weeks old C57Bl/6J mice at the time point of 8 weeks after OVX as described by Zhou and colleagues [53]. Additionally, the skeletal response to OVX was detected already 1 month after OVX surgery [54], demonstrating that our study design is suitable for comparing the influence of sensory and sympathetic neurotransmitters on fracture healing under quasi menopausal conditions.

## 4. Materials and Methods

### 4.1. Animals

All experiments were conducted in accordance with the local veterinary administration (Umweltamt, Dept. Veterinärwesen und Verbraucherschutz, City Regensburg, approved on 19.12.2014) and in agreement with the ethical committee appointed by the local authority (Regierung Unterfranken, Bayern, Germany) controlling animal experimental usage (Az.: 54-2532.1-23/14). Experimental protocols and methods were approved and carried out according to the regulations and guidelines of these committees. We used 10 weeks old female mice [53,55,56], kept under standard housing conditions (12h dark/light cycle) with free access to food and water. We used Tac1-/- mice that do not produce SP due to a targeted mutation in the Tachykinin 1 gene [57]. In addition, we used α-CGRP-/- mice (generous gift from Ronald B. Emeson; Department of Pharmacology, Molecular Physiology, and Biophysics, Vanderbilt University School of Medicine, Nashville, TN, USA), created by an insertion of a stop-codon into the α-CGRP coding region [58]. Both strains were bred back to a C57Bl6/J background. C57Bl6/J mice (Breeding pairs: Charles River, Sulzfeld, Germany; breeding with offspring: central animal facility, University of Regensburg, Regensburg; barrier-maintained mouse colonies) were randomly divided into a control group and a sympathectomized group. 6-hydroxydopamine (6-OHDA, chemical sympathectomy using 80 µg/g bodyweight, Sigma, Steinheim, Germany) was injected i.p. at days 8, 7, and 6 before and on day 13 after (in mice used for analysis of time points 16 and 21 days after fracture) fracture setting in a group of C57Bl6/J mice to destroy peripheral sympathetic nerve fibers, thereby reducing the production of adrenergic neurotransmitters by about 80% [20,21].

### 4.2. Operation Procedures

General anesthesia was performed in both procedures—OVX and femur fracturing—using a combination of fentanyl (0.05 mg/kg; JANSSEN-CILAG GmbH, Neuss, Germany), midazolam (5 mg/kg; ratiopharm GmbH, Ulm, Germany) and medetomidine (0.5 mg/kg; Domitor, Orion Corporation, Espoo, Finland). All mice underwent bilateral ovariectomy (OVX) at the age of 9–10 weeks by ligation and removal of the ovaries [59]. Directly after OVX, mice received a buprenorphine hydrochloride injection (0.1 mg/kg, SC; Buprenovet, Bayer Vital GmbH, Leverkusen, Germany) for analgesia. Femoral fractures were set 28 days after OVX using a standardized mid-diaphyseal fracture model. The fractures were stabilized intramedullarly by using an injection needle (24G, B. Braun Meslungen AG, Melsungen, Germany) serving as a nail [4,60] (Niedermair et al. 2014; Holstein et al. 2007). A fracture machine was used for fracturing left femora by using three-point bending (modified from protocol by Bertrand et al. 2013 [61]). Mice were divided into 5 groups, according to the analyzed time points after fracture (5, 9, 13, 16, and 21 days post-fracture). At these specific time points, locomotion analysis and the Dynamic Plantar Aesthesiometer test (Ugo Basile, Comerio, Italy) were conducted. Afterwards, mice of the respective group at that time point (time points after fracture: 5, 9, 13, 16, and 21 days post-fracture) were anesthetized and periorbital blood was collected [62]. Afterwards, mice were euthanized by using cervical dislocation.

### 4.3. Locomotion Analysis

Differences in locomotion were analyzed 2 days before, and 5, 9, 13, 16, and 20 days after setting the fractures. Mice were set separately into new home cages and monitored with a video camera for 1 h during night time (9:30–10:30 p.m.) after an appropriate acclimatization period of 2–3 h. Total distance moved (cm) was analyzed using the EthoVision XT 9 Video-Tracking-System (Noldus Information Technology, Wageningen, The Netherlands).

### 4.4. Dynamic Plantar Aesthesiometer Test

Mechanical hyperalgesia (touch sensitivity) was analyzed in both hind legs on days -4 and -2 before, and on days 5, 9, 13, 16, and 21 after fracture, using the Dynamic Plantar Aesthesiometer test (Ugo Basile, Comerio, Italy) as described before [4]. Briefly, the mice were placed on a grid in separate areas and the measurement device was placed under one paw. Upon starting, the automatic process raised a von Frey filament (part of the Dynamic Plantar Aesthesiometer device; Ugo Basile, Comero Italy) with steadily increasing force until the mouse withdrew the paw. Three values were recorded in each test for each hind paw and were averaged.

### 4.5. Sample Preparation for Histological Stainings

Femora were dissected 5, 9, 13, 16, and 21 days after fracture and prepared for histological staining. In brief, bones were fixed in 10% paraformaldehyde (Merck Chemicals GmbH, Darmstadt, Germany) (in PBS) for 24 h and decalcified for 6 weeks in 20% ethylene diaminetetraacetic acid (EDTA, pH 7.3; Roth, Karlsruhe, Germany). The intramedullary pin was removed and the bone was dehydrated through a graded series of ethanol solutions. Afterwards, femora were embedded into paraffin and 5 µm sections were cut along the shaft axis. Three sagittal paraffin sections with a minimal intersection distance of 30 µm were selected for all stainings, respectively, and were deparaffinized and rehydrated before the staining procedures. After staining, overview images were scanned for further semi-quantitative analysis (TissueFAXSi plus System, TissueGnostics, Vienna, Austria; INST89/341-1FUGG).

### 4.6. Alcian Blue—Sirius Red Staining

For morphometric analysis of mesenchymal, cartilaginous and bony callus tissue, sections were stained with alcian blue and sirius red. Deparaffinized and rehydrated sections were stained for 15 min with Weigert’s Hämatoxylin (Roth, Karlsruhe, Germany). Then, slides were washed with distilled water (ddH_2_O) for 5 min and with tap water for 10 min. Sections were treated with 3% acetic acid (pH 2.5; Roth, Karlsruhe, Germany) for 3 min and stained with 1% alcian blue (in 3% acetic acid, filtered; Sigma-Aldrich Chemie GmbH, Taufkirchen, Germany) for 30 min. Afterwards, sections were washed with tap water for 10 min and stained for 60 min in 0.1% (*w*/*v*) sirius red (Chroma, Münster, Germany; in saturated aqueous solution of picric acid, pH 2.0; AppliChem GmbH, Darmstadt, Germany). Afterwards, slides were incubated in 0.01 M HCL (Fluka, Honeywell Specialty Chemicals Seelze GmbH, Seelze, Germany) for 2 min and dehydrated in an ascending ethanol series. Finally, sections were incubated in xylol for 10 min 2 × times, and were mounted with Depex^®^ (Serva, Electrophoresis GmbH, Heidelberg, Germany) for microscopy.

### 4.7. Collagen X (Col X) Staining for Detection of Hypertrophic Chondrocytes

Col X positive area was stained with a mouse anti-col X antibody (# 14-9771-82, diluted 1:50; Invitrogen, ThermoFisher Scientific, Carlsbad, CA, USA) together with the DAKO^®^ Animal Research Kit (Dako North America, Inc., Carpinteria, CA, USA) as described before [4]. Staining of the growth plate served as a positive control and an isotype antibody (# ab18443, Abcam, Cambridge, UK) was used as a negative control.

### 4.8. Staining of Tartrate Resistant Acid Phosphatase (TRAP)-Positive Cells

Staining was performed using the Acid Phosphatase, Leukocyte Kit (387A-1KT, Sigma-Aldrich, Taufkrichen, Germany) according to the manufacturer’s instructions.

### 4.9. Immunohistological Staining for CD4, CD8, F4/80, iNos and Arg1

For antigen retrieval, deparaffinized and rehydrated femur sections were incubated in citrate buffer (pH 6.0) at 60 °C for 24 h. Slides were then washed with TBS buffer (+ 0.1% Tween; Sigma-Aldrich Chemie GmbH, Taufkirchen, Germany). Peroxidase was blocked in 3% hydrogen peroxide (Roth, 30% ROTIPURAN^®^, Karlsruhe, Geramny) for 10 min at RT. Afterwards unspecific binding was blocked in 5% goat serum (in TBS buffer + Tween) for 1 h at RT. Then, the following primary antibodies were incubated over night at 4 °C: CD 4—1:100 (# 25229, Cell Signaling Technology (CST), Danvers, MA, USA), CD 8—1:400 (# 98941, CST), F4/80—1:250 (# 70076, CST), iNos—1:75 (# 13120, CST), Arg1—1:100 (# 93668, CST) or the appropriate concentration of the isotype control (# ab172730, Abcam, Cambridge, UK) in antibody diluent (# 8112, SignalStain^®^ Antibody Diluent, CST). Sections were washed three times with TBS buffer + 0.1% Tween. For primary antibody detection, the SignalStain^®^ Boost IHC Detection Reagent (# 8114, CST) was applied for 30 min at RT. Then the slides were washed 3 × times with TBS + 0.1% Tween. Afterwards sections were incubated with DAB substrate for 10 min. Slides were washed 2 × times in ddH2O, nuclei were stained with hematoxylin (# 1.05174, Merck, Darmstadt, Germany) and blued in tap water for 30 min. Finally, sections were mounted with Roti-Mount Aqua (Roth, Karlsruhe, Germany) for microscopy.

### 4.10. Serum Analysis

Collected blood was kept at RT for up to 60 min to allow clotting. Afterwards, samples were centrifuged at 2000× *g* for 10 min to prepare serum. Serum samples were stored at −80 °C until usage.

Cross Linked C-Telopeptide of Type I Collagen (CTX I) was analyzed as a bone resorption marker in serum 9 and 16 days after fracture by ELISA (# CEA665Mu, Cloud-Clone Corp.; Biozol Diagnostica Vertrieb GmbH, Eching, Germany) according to the manufacturer’s instructions.

The QuantiChrom™ Alkaline Phosphatase Assay Kit (# DALP-250, BioAssay Systems, Biotrend Chemikalien GmbH, Cologne, Germany) was used according to the manufacturer’s protocol to measure alkaline phosphatase (ALP) in serum as a marker for bone formation.

### 4.11. Statistics

Graph Pad Prism 6.0 software (GraphPad Software, San Diego, CA, USA) was used for statistical analysis and Graph preparation. Data shown as time course are expressed as mean ± SEM or SD as indicated in the figure legend. Data represented in box plots are expressed as median ± min/max. Differences between the groups were analyzed using a two-tailed Mann-Whitney *U*-test. *p*-Values less than 0.05 were considered as significant.

## 5. Conclusions

In conclusion, the absence of α-CGRP seems to promote M2 macrophage polarization and reinforced pain-related processes, but has no effects on fracture callus maturation and the T-cell number in OVX mice as a model for impaired musculoskeletal conditions. The absence of SP seems to modulate pain-related processes and locomotion activity and affects ossification of hypertrophic cartilage tissue. The destruction of sympathetic nerve fibers has no effect on pain-related processes, but seems to influence locomotion activity and bony callus maturation, and modulates the CD4/CD8 T-cell ratio.

Furthermore, our data indicate stronger effects of SP and sympathetic nerve fibers on callus maturation during middle and late stages of fracture healing, whereas loss of α-CGRP might influence macrophage-driven inflammatory processes during the early stages under experimental postmenopausal conditions. We suggest that SP and sympathetic signals play different roles in bone regeneration under physiological versus pathophysiological conditions.

## Figures and Tables

**Table 1:** Serum analysis of bone resorption marker CTX I and bone formation marker ALP. CTX I and ALP were determined 9and 16 days after fracture in serum of WT, Tac1−/−, α-CGRP−/−, and SYX mice. Data are expressed as mean ± SD. N = 4–5. **^+^** indicates difference compared to WT with *p* = 0.0649.

Serum Marker	CTX I (µg/mL)		ALP (IU/L = µmol/(L*min))	
Time Point	Day 9	Day 16	Day 9	Day 16
Sample				
WT	5.035 ± 1.342	6.377 ± 1.208	269.5 ± 22.56	352.4 ± 47.82
Tac1−/−	6.707 ± 2.82	5.088 ± 1.257	275.5 ± 25.15	348.8 ± 64.69
α-CGRP−/−	8.238 ± 3.47	6.507 ± 0.497	244.6 ± 27.02	342.4 ± 73.57
SYX	5.638 ± 1.382	6.665 ± 1.841	291.6 ± 27.88	406.5 ± 36.28 ^+^

## Supplementary Materials

Supplementary Materials can be found at https://www.mdpi.com/1422-0067/21/2/405/s1.
