# Supplementary material for: Impact of the Sensory and Sympathetic Nervous System on Fracture Healing in Ovariectomized Mice
Source: Int J Mol Sci. 2020 Jan 8;21(2):405. doi: 10.3390/ijms21020405 (PMC7013559; doi:10.3390/ijms21020405)
Supplement: Supplementary file 1 [file ijms-21-00405-s001.pdf]

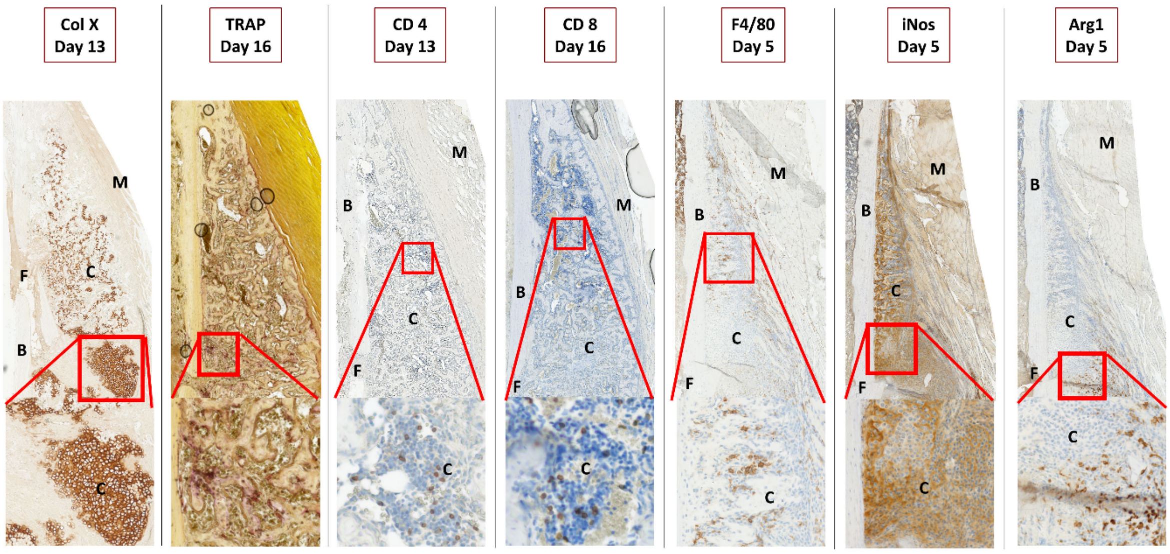

Supplementary Figure 1: Representative images of histological and immunohistological stainings in callus tissue from WT control animals – comparable to the representative images shown in Figures 5, 6 and 7. Overview images were scanned with 20-fold magnification (TissueFaxSI plus). Red boxes demonstrate the enlarged view. B=bone, C=Callus tissue, BM=bone marrow, F=Fracture site, M=Muscle.
